# Kidcope and the COVID-19 Pandemic: Understanding High School Students’ Coping and Emotional Well-Being

**DOI:** 10.3390/ijerph181910207

**Published:** 2021-09-28

**Authors:** Wan-Jung Hsieh, Tara Powell, Kevin Tan, Jun-Hong Chen

**Affiliations:** 1 School of Social Work, University of Illinois at Urbana-Champaign, Urbana, IL 61801, USA; tlpowell@illinois.edu (T.P.); kevintan@illinois.edu (K.T.); 2 Brown School, Washington University in St. Louis, St. Louis, MO 63130, USA; jun-hongchen@wustl.edu

**Keywords:** COVID-19 pandemic, emotional well-being, mental health, coping, high school, Kidcope

## Abstract

The COVID-19 pandemic has resulted in social isolation, grief, and loss among many adolescents. As the pandemic continues to impact individuals and communities across the globe, it is critical to address the psychological well-being of youths. More studies are needed to understand the effective ways adolescents cope with pandemic-related psychological distress. In this study, 146 students from 1 high school in a U.S. midwestern state completed an adapted version of Kidcope, a widely used coping instrument in disaster research, and measures were taken on generalized distress and COVID-19-related worries. Findings indicated that most students experienced COVID-19-related fears and general emotional distress. Additionally, we found that disengagement coping strategies were associated with lower general distress (*p* ≤ 0.05) and COVID-19 worries (*p* ≤ 0.10). Active coping was not associated with general distress and COVID-19 worries. Overall, our findings highlight the need to develop tailored interventions targeting youth coping strategies to reduce and prevent emotional distress and amplify healthy coping skills as the pandemic persists.

## 1. Introduction

The Coronavirus-19 (COVID-19) pandemic has created an unpreceded global public health crisis. The United States (U.S.) is among the countries most impacted by the pandemic, leading the world in confirmed cases and COVID-19-related deaths. As of February 2021, there have been approximately 28 million confirmed cases in the United States and almost 500,000 deaths [1].

The pandemic has significantly impacted school-aged youth. Despite children and adolescents being less likely to present with COVID-19 symptoms compared to adults [2], many have suffered from indirect effects, such as social isolation, grief and loss, and separation from school, peers, extended families, and communities [3]. Since March 2020, many states in the U.S. have abruptly and rapidly implemented disease containment policies by closing schools, activity centers, and educational institutes in an effort to reduce the transmission rates of COVID-19, which affects nearly all of the 55 million students from across the nation [4]. As the pandemic continues to interrupt the consistency, routine, and structure of life, scholars and mental health professionals alike have raised serious concerns about the heightened rates of depression, anxiety, and distress associated with the pandemic among youths [5,6]. Thus, it is important to understand factors that influence distress among youths in order to reduce the likelihood of adverse mental health consequences during the pandemic and over its long-term recovery.

### 1.1. COVID-19 Pandemic and Youth Emotional Well-Being

Scholars project the COVID-19 pandemic will have long-term consequences for youths’ psychological and emotional well-being [7,8]. Adolescence is a critical developmental period in which young people experience tremendous growth [9,10,11]. Experiencing the pandemic during this life stage, however, can have detrimental consequences, especially if they are not equipped with the appropriate coping skills [12].

Although data are still emerging on pandemic-related psychological distress, studies have found associations between COVID-19-related stressors and adverse mental health outcomes among young people. Pandemic-related stressors have been associated with increased anxiety and depression among school-aged children and youth [5,7]. During the early stages of the pandemic, Xie et al. [13] observed that, after 34 days of home restriction, clinical levels of depression and anxiety symptoms were at a rate of 22.6% and 18.9% among Chinese school-aged children. Other studies exploring youth mental health during COVID-19 found clinical rates of depression as high as 43.7% and anxiety symptoms at 37.4%; these values are significantly elevated from pre-pandemic levels [14].

Social isolation and loneliness during the pandemic have been attributed to heightened levels of psychological distress [15]. Zhou and colleagues (2020) found that clinical levels of depression and anxiety remained high among adolescents, even after the infection rate of COVID-19 eased. Loades et al. [15] reported the duration of loneliness was associated with greater psychological distress, suggesting that continued quarantine and mitigation strategies will lead to sustained mental health challenges.

It is anticipated that COVID-19-related psychological distress among children and adolescents will continue beyond the pandemic. Long-lasting psychological effects among young people may be further exacerbated by pandemic-related factors such as lacking school connectedness, economic instability, preexisting health conditions, and domestic violence [3,4,16]. For example, school closures may affect students beyond education; other critical services such as nutrition supply and health/mental health services are disrupted by the prolonged closure policy [4]. Additionally, youths are more vulnerable to exposure to harsh parenting, abuse, or neglect within the family as a result of their parents’ emotional distress [3,17].

### 1.2. Coping and Disasters

The toll of COVID-19 on the psychological well-being of children and adolescents has been well documented, making it critical for scholars to assess contributors to pandemic-related distress. Research conducted after natural and human-made disasters has explored several factors that increase or reduce the likelihood of distress symptoms (i.e., PTSD, depression) among young people. Scholars have consistently found associations between the way a young person copes and disaster-related distress. Negative or maladaptive strategies such as rumination, escape-oriented, and avoidant coping have been associated with greater symptoms of depression, anxiety, and post-traumatic stress [18,19]. Conversely, adaptive coping strategies such as problem-focused, positive cognitive restructuring, and use of emotional support have all been found to buffer post-disaster psychopathology [20,21,22].

Emerging research also suggests that coping styles influence COVID-19-related psychological challenges among children and youth. Liang et al. (2020) examined coping among 584 youths two weeks after the start of the pandemic in China, reporting that a higher use of negative strategies (e.g., using alcohol to reduce stress) was associated with greater psychological distress, whereas active coping (e.g., trying to see things in a positive light) was related with fewer mental health symptoms [23]. Another study of Chinese youths during the pandemic by Duan et al. (2020) demonstrated that emotion-focused coping (e.g., avoiding activities to manage difficulties) was related to greater rates of depression, whereas problem-focused coping (e.g., positive behavioral strategies to handle stress) was inversely related to depression symptomatology [5]. Domínguez-Álvarez et al. (2020) assessed coping strategies among pandemic-affected Spanish children and adolescents, finding that disengagement coping (e.g., disengaging or escaping from the source of stress) predicted higher emotional and behavioral difficulties, whereas engagement coping (e.g., acceptance, positive thinking) was associated with the greater psychological adjustment such as social bonding and prosocial behaviors [24].

Scholars have begun to explore the association between coping and COVID-19-related psychological symptoms; however, pandemic-related studies have primarily been conducted in China and Europe. While a vast body of literature exists on the relationship between young people’s psychological distress and coping, particularly during the recovery period from a crisis [19,25,26], scholars have yet to explore coping in the prevailing pandemic among children and youths in the United States. This study utilizes Kidcope, a widely used coping measure to explore the relationship between coping and emotional distress among high school students within a United States midwestern community during the COVID-19 pandemic.

### 1.3. Study Hypothesis

This study is guided by the following research questions: (1) What dominant coping strategies were employed among a sample of adolescents in the United States (U.S.) at the onset of the COVID-19 pandemic? (2) What are the relationships between coping strategies and emotional distress among U.S. adolescents? Specifically, we hypothesize that adaptive or active coping will protect against emotional distress symptoms [20,21,22]. On the contrary, we believe that the utilization of disengagement coping will serve as a maladaptive coping strategy and be associated with higher distress symptoms among youths [18,19].

## 2. Methods

### 2.1. Setting

All 9th–11th grade high school students in one United States midwestern high school were invited to participate in this study. The high school consists of approximately 900 students. The school’s demographics are 87% White, 4.7% Hispanic, and 2.1% Asian. Furthermore, 14.0% of students have an individualized learning plan (IEP) and 16.6% are considered low-income. The district high school graduation rate stands at 95%. The 2018 median household income from the U.S. Census Bureau of the school enrollment area is USD 115,619 which is comparatively higher than the overall U.S. at USD 63,170.

The state ordered schools to rapidly close in March 2020 to manage the COVID-19 pandemic. Within a week, the school adopted an online learning system and students had to socially distance and remained in their own homes. All school-related extracurricular and community social events were canceled. In the last week of the academic year (end of May), all 9th–11th grade students were invited to participate in this study through an email sent from the district office. This email contained the Qualtrics survey link, an invitation letter from the study’s third author, and the study’s consent form. Parents were informed via email a week before the survey. Students were provided with a letter seeking assent. Students completed the survey only if they provided consent. No compensation was provided for completing the survey. The entire survey took approximately 20 min to complete and consisted of quantitative and open-ended qualitative responses. The 60 questions on the survey inquired about personal and familial experience with COVID-19, lifestyle changes, coping strategies, experience and perception of remote teaching, and students’ social and emotional needs. The University (Blinded for Peer Review) Institutional Review Board approved the research procedures.

### 2.2. Measures

COVID-19 worries is one of two measures of distress used in this study. It was assessed based on 4 questions from the Coronavirus Health Impact Survey (CRISIS), which is funded by the National Institute of Mental Health, the Child Mind Institute, and the Nathan Kline Institute. The questionnaires are publicly available at www.crisissurvey.org. The questions asked how worried respondents were about “…being affected?”, “…friends or family being affected?”, “…your physical health being influenced by COVID-19”, and “…your mental/emotional health being influenced by COVID-19?” Respondents responded to a five-point scale—“1” = “Not at all” to “5” = “Extremely”. A higher score indicated more COVID-19-related worries. Cronbach’s alpha in our study sample was α = 0.78.

General emotional distress is based on 10 items from the CRISIS survey instrument. These items are “how worried were you generally?”, “how happy verses sad were you?”, “how much were you able to enjoy your usual activities?”, “how relaxed versus anxious were you?”, “how fidgety or restless were you?”, “how fatigued or tired were you?”, “how well were you able to concentrate or focus?”, “how irritable or easily angered were you?”, “how lonely were you?”, and “to what extent did you have negative thoughts, thoughts about unpleasant experiences or things that make you feel bad?”. The survey asked about students’ experiences in the past 2 months. Students responded to each item on a 5-point scale. Two items in the original instrument were reverse coded so that higher values consistently indicated more negative emotions or worries. Cronbach’s alpha in our study sample was α = 0.86.

Coping Strategies. Coping strategies were assessed using the Kidcope scale [27,28]. Kidcope has been applied in multiple studies to assess adaptive/positive and maladaptive/negative coping strategies used by children and adolescents, aged between 7 and 18 years [18,27,29]. It is one of the most widely used measures in post-disaster literature and exists in several versions that range from 10 to 15 items exploring young people’s ways of coping, and how those relate to physical and mental health problems and psychological adjustment [30,31,32]. Kidcope has also been applied to understanding young people’s coping in non-disaster stressors such as physical illness, HIV, interpersonal violence, and school-related stress [27,29,33].

In our study, we used a modified version of Kidcope to assesses active and disengagement coping [27,34,35]. Active coping was based on three items: “I tried to fix the COVID-19 problem by thinking of answers”, “I tried to address the COVID-19 problem by doing something or talking to someone”, and “I tried to calm myself down”. We also examined Disengagement coping, which is based on four items: “I just tried to forget about the COVID-19 pandemic”, “I did something like watch TV or played a game to forget it”, “I kept quiet about the COVID-19 problem”, and “I did not do anything because the COVID-19 problem could not be fixed”. Students were asked how much each of these strategies helped and they responded over a three-point scale: “1” = “Not at all”, “2” = “A Little”, and “3” = “A lot”. Both measures reflected adequate internal consistency in our sample (active coping Cronbach’s alpha = 0.70 and disengagement coping Cronbach’s alpha = 0.64).

Covariates included grade-level (9th, 10th and 11th grade), social-economic status (“1” if respondents received financial aid 3 months before the COVID-19 crisis; “0” if none), self-reported physical health (“1” = “Excellent” to “5” = “Poor”), self-reported mental/emotional health before the COVID-19 crisis (“1” = “Excellent” to “5” = “Poor”), and family impact of COVID-19. Family impact on family members is based on respondents’ reports that they had a family member who had been diagnosed with COVID-19, fallen ill physically, been hospitalized, self-quarantined with symptoms, self-quarantined without symptoms (due to possible exposure), lost or been laid off from a job, a reduced ability to earn money, and/or passed away as a result of COVID-19. A higher value indicated the greater family impact of COVID-19.

## 3. Analyses

We first conducted frequency analyses examining the mean of individual COVID worries and general emotional distress items. The frequency of how often youths used each of the coping strategies and their perceived effectiveness was also assessed. Bivariate correlations were also performed to examine the relationship between individual coping items, COVID, and general emotional distress. Frequency and correlation analyses were conducted in SPSS v27 [36].

**Figure 1:**
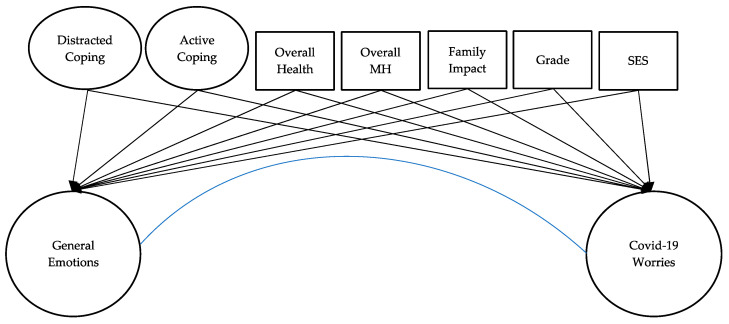
Analysis framework.

A structural equation model (SEM) within Mplus v8.1 was subsequently applied to investigate the relationships among child coping strategies (active coping and disengagement coping) and mental health (general emotional distress and COVID-19 worries) while taking into account covariates, including grade, self-reported physical health, self-reported mental/emotional health before the COVID-19 crisis, social-economic status, and family impacts of COVID-19 (e.g., fallen ill physically, been hospitalized, or self-quarantined with symptoms). Our SEM analysis model is presented in Figure 1. For each construct, we modeled a direct pathway with our covariates to reflect its direct impact on coping and students’ mental health. We examined changes in the coefficients among our constructs with the inclusion of each covariate.

In our SEM model, we examined the following model fit indices [37]: model chi-square, the comparative fit index (CFI), the Tucker–Lewis index (TLI), and the root mean square error of approximation (RMSEA). The chi-square model assesses the fit between the data from the set of measurement items and the hypothesized model, with an insignificant chi-square value indicating that the model is consistent with the data [38]. The CFI and TLI are incremental fit indices that compare the fit of a hypothesized model with that of a baseline model; values greater than 0.90 are considered a good fit [39]. The RMSEA is a measure of how close the implied matrix is to the observed variance–covariance matrix; values less than 0.05 are considered evidence of a good fit, values between 0.05 and 0.08 indicate a fair fit, and values greater than 0.10 represent a poor fit [40]. Missing data were handled using a full information maximum likelihood (FIML) estimation approach which is the default function in Mplus.

## 4. Results

Among the sample of 146 students, most of the participants were in 11th grade (53%), followed by 10th and 9th grade at 32% and 12%, respectively. Most participants were female students, (n = 113, 77%), and 25% (n = 25) reported their family received financial assistance, such as free and/or reduced-price lunch, aid to families with dependent children, general assistance, or temporary assistance for needy families in the 3 months prior to the COVID-19 crisis. Regarding the impact of COVID-19 on participants’ families, 27% (n = 51) of participants reported having to self-quarantine (with and without symptoms) and 26% (n = 49) reported the pandemic caused job loss and reduced income.

The most common COVID-19-related worries included mental health being impacted by the pandemic (M = 2.88, SD = 1.38) and family or friends being affected (M = 2.75, SD = 1.18). Among the general emotional distress items, enjoying usual activities, feeling unfocused/ distracted, feeling anxious, and having negative thoughts demonstrated the highest intensity among the participants with the average scores at M = 3.44 (SD = 0.97), M = 3.38 (SD = 1.19), M = 2.98 (SD = 1.18), and M = 2.88 (SD = 1.27), respectively, on a five-point scale. Table 1 summarizes demographic characteristics and means of individual items assessing general emotional distress and COVID-19 worries.

Among three active copings strategies, “I tried to calm myself down” was not only the most applied strategy (n = 68, 46.9%) among participants, but was also perceived as the most effective, with a mean of 2.11 (SD = 0.71). Among the four disengagement coping strategies, “I did something like watch TV or played a game to forget it” was both the most applied coping strategy (n = 76, 52.4%), as well as perceived as the most effective, with a mean of 2.28 (SD = 0.71). Frequencies and means of individual coping items are presented in Table 2.

Correlation results (Table 3) indicated that COVID-19 and general emotional distress were inversely correlated with two active coping items. Regarding COVID worries, coping item six, “addressing the pandemic by talking to someone” *r* = −0.26, *p* = 0.01, and item seven, “Trying to calm myself down” *r* = −0.40, *p* = 0.001, indicated significant inverse relationships. These two strategies were also inversely correlated to general emotional distress, illustrating significant relationships between coping item six *r* = −0.29, *p* = 0.01, and coping item seven *r* = −0.31, *p* = 0.001, respectively.

### SEM Model

The SEM analyses illustrated a significant relationship between disengagement coping and lower general emotional distress and COVID-19 worries. Specifically, a one-unit increase in disengagement coping was associated with a significant decrease in general emotional distress by a 0.442 unit while holding other variables constant (*p* ≤ 0.05). Additionally, a one-unit increase in disengagement coping was associated with a marginally significant decrease in COVID-19 worries by a 0.777 unit while holding other variables constant (*p* ≤ 0.10). Active coping was not significantly associated with general emotional distress and COVID-19 worries. Covariates (i.e., the grade of the youth, socioeconomic status, family impact, overall mental health, and physical health) had no significant associations with coping strategies

A significant association between COVID-19 worries and general emotional distress (*p* < 0.05) was also detected. A one unit increase in general emotional distress was associated with higher COVID-19 worries by 0.213. Additionally, those who experienced a greater family impact reported more general distress. Specifically, a one unit increase in family impact was associated with a 0.168 increase in general emotional distress.

Fit indices indicated that the model marginally fitted the data well (CFI = 0.855; TLI = 0.827; RMSEA = 0.062; *p*-value of Chi-square test less than 0.05). SEM results are presented in Table 4.

## 5. Discussion

The present study is among the first to examine the relationship between coping and emotional distress among a sample of U.S. high school adolescents during the COVID-19 pandemic. Findings indicated that most youths experienced worries about their friends, families, and their own physical health being affected by COVID-19 during the pandemic. Additionally, feeling unfocused, anxious, and having negative thoughts were among the most common general distress symptoms experienced by youths in the study. These findings corroborate other studies that adolescents have experienced heightened levels of anxiety, COVID-19-related fears, and generalized distress during the pandemic [6,7].

We also found that disengagement coping was associated with lower general emotional distress, whereas active coping had no effect on psychological outcomes. Coping is a complex construct that can play a significant role in protecting against or increasing the risk of adverse mental health outcomes during stressful life experiences. Studies during the COVID-19 pandemic have consistently documented that youths who employ maladaptive or avoidant coping behaviors such as distraction, self-blame, or behavioral disengagement are at greater risk for emotional distress, whereas those who utilize active/adaptive coping strategies are less likely to experience adverse mental health symptoms [41,42]. Our findings are contrary to research indicating disengagement is a maladaptive strategy that increases the risk of psychological distress, and active coping protects against adverse mental health outcomes [43,44].

Several reasons may account for our inconsistent findings. The active coping questions in our measure included *primary control coping*, which entails addressing the stressor by employing strategies such as problem solving or emotional expression. Conversely, disengagement items included the use of self-distraction, which is considered a secondary control coping strategy [42]. While scholars have consistently found that the use of primary control coping is related to better psychological outcomes, these associations generally only exist in controllable situations/contexts [45]. Alternatively, scholars have noted that secondary control strategies may be more effective in an uncontrollable context/situation [45,46]. The global scale of the COVID-19 spread may foster a sense of incapability among young people to alter the situation, thus resorting to secondary control strategies such as distraction to cope with the effects of the pandemic. Contrary to the expectations in the literature, actively coping with the pandemic by thinking of answers may not be as effective given the uncontrollability of COVID-19. Considering the uncertain nature of the pandemic, the use of disengagement appeared to play a protective role against emotional distress among our sample.

Cultural and contextual differences may also account for inconsistent coping outcomes in our sample compared to other studies during the pandemic. Most research examining adolescent coping during the pandemic has been conducted in Europe or Asia; therefore, cultural differences in the way a young person copes may exist. For example, Orgilés et al. [47] examined coping behaviors among youths in three European countries, finding that strategies varied by country. Scholars have noted that coping with extreme stressors varies by culture; however, these differences have yet to be extensively studied [48]. The pandemic also triggered containment measures in European and Asian countries months before the United States. Our study was conducted only a few months into stay-at-home orders in the U.S.; therefore, disengaging from pandemic-related stressors may have been an effective way to cope initially, but it is unknown whether this way of coping would continue to buffer against distress symptoms. Considering cultural and contextual factors associated with coping, continued research across cultures and throughout the duration of the pandemic is warranted.

The socio-economic status of respondents is another important consideration. A study by Domínguez-Álvarez’s et al. [24] on Spanish middle-aged children found an association between disengagement coping and poorer mental health symptoms. However, it is observed that half of the parents in this study were reportedly financially impacted by the pandemic. This is in stark contrast with our study’s population in which only a quarter of our sample’s parents were financially burdened by the pandemic. Notably, our study is based on a community in which median household income is almost two times higher than the overall U.S. population (USD 115,619 versus USD 63,170). Youths in our study may have the luxury of family resources to ride out the pandemic, and they may have more access to material items (e.g., computer gaming) to enable the use of disengagement through distraction compared to the study population in Alvarez et al. [24]. The ever-growing body of pandemic literature drawing attention to the association between socio-economic status and health outcomes [49,50,51] highlights the need to consider the social context of our sample in comparing our findings with prior literature. To achieve a holistic understanding of the relationship between coping and psychological distress, it is essential to explore the context of the stressor and the role of the coping response.

### 5.1. Implications

The current study addressed the timely and important topic of adolescents’ emotional well-being and coping during the ongoing pandemic. Several implications for practice and future research should be considered. First, as uncertainty and disruption continue, it is critical for accessible mental health resources and services. Our study illustrated that many participants experienced worries about how COVID-19 would impact their mental health, felt anxious, and had difficulties concentrating or focusing.

Interventions designed to reduce or prevent generalized distress and amplify healthy coping skills may be beneficial for youths who are experiencing the ongoing stress of the pandemic. Such interventions should be culturally and contextually applicable and may include activities to increase social support, which can reduce feelings of isolation and/or mindfulness to help young people self-regulate and calm themselves when experiencing feelings of anxiety [52]. These programs may also include parental psycho-educational material on how to reduce distress and build healthy coping strategies for their children. Given the social distancing requirements of the pandemic, remote approaches to engage youths are needed. Innovative ways of increasing social support and reducing psychological distress during the pandemic, such as the use of text-based services with older adults [53] and WebChat/email services with adolescents [54], have been shown to be successful in alleviating adverse mental health symptoms.

As the pandemic lingers on with concerns over new emerging variants, mental health service providers need to adapt and find creative ways to provide mental health supports and services for youths.

### 5.2. Limitations and Directions for Future Research

There are several strengths and limitations of the study that should be noted. This study is specific to a convenience sample of students from one high school in a predominantly white and affluent community in a U.S. midwestern state, and therefore cannot be generalized to the broader U.S. population. The majority of our respondents are females (close to 80%), which skews any comparison on coping and distress involving gender. Additional research is needed with more diverse samples to assess potential racial, gendered, and economic differences in outcomes.

Other limitations include the small sample size limiting the statistical power for our analysis and the cross-sectional nature of the data. We noted the marginal model fit results of our SEM model, which can be clarified with a larger sample [55]. We are also unable to make strong causal inference over the direction that coping behaviors lead to emotional distress. We do not rule out the possibility that one’s emotional state could also influence one’s coping strategies. Additional research should draw on a larger sample size and assess the longitudinal relationship between coping behaviors and emotional distress across the duration of the pandemic.

The use of self-report measures was another limitation to our study. Given the individualized nature of coping and distress, self-reporting can provide important information about a youth’s internal experience. The use of additional reporters (e.g., parents, teachers), however, could provide a more holistic understanding of observable coping behaviors and distress symptoms that may not be captured in self-report measures. A final limitation was the use of Kidcope as a measure of coping. Kidcope is one of the most widely used coping measures in disaster research; however, it has yielded inconsistent factor structures in previous studies [56]. While the subscales of the Kidcope used in this study yielded adequate reliability, future research should examine the psychometric qualities of the measure in the context of the COVID-19 pandemic.

## 6. Conclusions

The COVID-19 pandemic continues to have a global impact, making it critical to address the emotional needs of young people. Our study illustrated that many youths experienced emotional distress and pandemic-related worries; however, effective coping strategies buffered the impact of these psychological distress symptoms. As COVID-19 disrupts communities across the globe, continued research and understanding of effective coping is crucial to reduce the short- and long-term psychological impact of the pandemic among young people.

## Figures and Tables

**Table 1:** Descriptive characteristics.

	N (%)
**Grade**	
9th Grade	17 (11.6)
10th Grade	51 (32.2)
11th Grade	77 (53.1)
**Gender**	
Female	113 (77.3)
Male	33 (22.6)
**Received Financial Assistance**	
Yes	25 (17.1)
No	116 (79.5)
**Family Impact of COVID-19**	
Fallen physically ill	8 (4.3)
Hospitalized	1 (0.5)
Put into self-quarantine with symptoms	10 (5.4)
Put into self-quarantine without symptoms	41 (21.9)
Lost or been laid off from job	13 (7.0)
Reduced ability to earn money	36 (19.3)
Passed away	1 (0.5)
None of the above	77 (41.2)
	**M (SD)**
**COVID Worries**	
Being affected	2.15 (0.99)
Friends or family being affected	2.75 (1.18)
Physical health influenced by COVID-19	1.92 (1.03)
Mental health influenced by COVID-19	2.88 (1.38)
**General Emotional Distress**	
How worried were you generally	2.28 (0.98)
How happy verses sad were you	3.07 (0.99)
How much you enjoy your usual activities	3.44 (0.97)
How relaxed versus anxious were you	2.98 (1.18)
How fidgety or restless were you	2.54 (1.19)
How fatigued or tired were you	2.56 (1.24)
How well you are able to concentrate or focus	3.38 (1.42)
How irritable or easily angered were you	2.79 (1.26)
How lonely were you	2.69 (1.16)
How often did you have negative thoughts	2.88 (1.26)
**Mental Health Prior to COVID-19**	2.85 (1.19)
**Overall Physical health**	2.27 (0.96)

**Table 2:** Frequencies and means of use and perceived effectiveness of coping items.

	Used Strategy	Effectiveness
	N (%)	M (SD)
**Disengagement Coping**		
1. Tried to forget about the pandemic	46 (31.7)	1.85 (0.69)
2. Did something to forget it like watch TV/play video games	76 (52.4)	2.28 (0.64)
3. Kept quiet about the pandemic	29 (20.0)	1.78 (0.77)
4. Did nothing	45 (31.0)	1.70 (0.77)
**Active Coping**		
5. Tried to fix problem by thinking of answers	12 (8.3)	1.63 (0.79)
6. Address pandemic by talking to someone	36 (24.8)	1.88 (0.70)
7. Tried to calm self-down	68 (46.9)	2.11 (0.71)

**Table 3:** Bivariate Pearson’s R correlations of variables.

Variables	1	2	3	4	5	6	7	8	9
**Disengagement Coping**									
1. Coping: tried to forget	–								
2. Coping: watch TV	0.31 **	–							
3. Coping: kept quiet	0.23 *	0.16	–						
4. Coping: did nothing	0.18	0.17	0.05	–					
**Active Coping**									
5. Coping: fix problem	0.07	−0.10	−0.26 *	−0.17	–				
6. Coping: talk to someone	−0.02	0.03	−0.07	−0.27 *	0.25 *	–			
7. Coping: calm self	−0.03	0.25 *	−0.20	−0.14	0.23 *	0.27 **	–		
**Mental Health**									
8. COVID worries	−0.08	−0.07	0.14	−0.02	−0.06	−0.26 *	−0.40 **	–	
9. General emotional distress	−0.13	−0.13	0.06	−0.02	−0.07	−0.29 *	−0.31 **	0.59 ***	–

Note: * *p* ≤ 0.05, ** *p* ≤ 0.01, *** *p* ≤ 0.001.

**Table 4:** SEM model on coping strategies and mental health.

Main Predictors	Outcomes	
	General Emotional Distress	COVID-19 Worries
Active coping	0.054 (0.070)	0.115 (0.061)
Disengagement coping	−0.442 * (−0.451)	−0.777 ^†^ (−0.322)
**Model Fit Indexes**		
Chi-Square Test	*p* < 0.05	
RMSEA	0.062	
CFI	0.855	
TLI	0.827	
SRMR	0.096	

Note. * *p* ≤ 0.05 ^†^ *p* ≤ 0.10 Standardized coefficients are shown in parentheses. The model was performed by including the following covariates: Grade, SES, mental health, physical health, and family impact.

## Data Availability

The data presented in this study are available on request from the corresponding author. The data are not publicly available due to ethical restrictions.
